# Walkability is Only Part of the Story: Walking for Transportation in Stuttgart, Germany

**DOI:** 10.3390/ijerph110605849

**Published:** 2014-05-30

**Authors:** Maren Reyer, Stefan Fina, Stefan Siedentop, Wolfgang Schlicht

**Affiliations:** 1 Department of Sport and Exercise Science, Chair Exercise and Health Sciences, University of Stuttgart, Nobelstraße 15, D-70569 Stuttgart, Germany; E-Mail: wolfgang.schlicht@inspo.uni-stuttgart.de; 2 Institute of Regional Development Planning, University of Stuttgart, Pfaffenwaldring 7, D-70569 Stuttgart, Germany; E-Mail: stefan.fina@ireus.uni-stuttgart.de; 3 ILS—Research Institute for Regional and Urban Development, Bruederweg 22-24, D-44135 Dortmund, Germany; E-Mail: stefan.siedentop@ils-research.de

**Keywords:** physical activity, health, walkability, Walk Score, environment

## Abstract

In modern Western societies people often lead inactive and sedentary lifestyles, even though there is no doubt that physical activity and health are related. From an urban planning point of view it would be highly desirable to develop built environments in a way that supports people in leading more active and healthy lifestyles. Within this context there are several methods, predominantly used in the US, to measure the suitability of built environments for walking and cycling. Empirical studies show that people living in highly walkable areas are more physically active (for example, walk more or cycle more). The question is, however, whether these results are also valid for European cities given their different urban planning characteristics and infrastructure standards. To answer this question we used the Walkability-Index and the Walk Score to empirically investigate the associations between walkability and active transportation in the city of Stuttgart, Germany. In a sample of household survey data (*n* = 1.871) we found a noticeable relationship between walkability and active transportation—the more walkable an area was, the more active residents were. Although the statistical effect is small, the health impact might be of relevance. Being physically active is multi-determined and not only affected by the walkability of an area. We highlight these points with an excursion into research that the health and exercise sciences contribute to the topic. We propose to strengthen interdisciplinary research between the disciplines and to specifically collect data that captures the influence of the environment on physical activity in the future.

## 1. Introduction

There is convincing evidence that physical activity prevents the onset of cardio-metabolic risks and diseases [1]. Despite this convincing and strong evidence, most people in Europe and in other Western societies practice a sedentary and inactive lifestyle [2]. Physical activity is multi-determined behavior, which can only be understood if the interaction between personal and environmental determinants is considered, as is done in socio-ecological models [3]. Personal determinants are, for example, psychological concepts like attitudes, motives or volitional skills; environmental determinants are, for example, features of the built, technical and the social environment.

In exercise and health sciences there is a growing amount of work focusing on the built environment as an important meaningful determinant of persons’ physical activity [4]. The keyword in this work is walkability. This is defined in different disciplines in different manners. Its essence is defined here as “the extent to which the built environment is walking-friendly” [5]. From a transportation research and urban planning perspective, walkability is relevant in order to reduce traffic congestion and improve air quality. Public health researchers are interested in highly walkable neighborhoods because they assume an impact on active transportation, hence their support of active living in general. 

So far walkability has predominantly been an issue in Northern American and Australian research, but might be a promising approach for European cities as well. Although it might be difficult to use the same measurements for Europe’s more historical cities, with their more heterogeneous layout than that typical to the US with their traditionally more separated land-use patterns, our hypothesis states that walkability issues are nevertheless present in the European city. However, measurement methods will have to be expanded to capture the spatial variations and come up with an enhanced walkability assessment. This article aims to assess the walkability of the city of Stuttgart, Germany using two different types of indicators: The Walkability-Index (WAI) [6] and the Walk Score [7]. Both were developed to map out high and low walkability areas. Data from a household survey were used to investigate the association between urban forms and active transportation, followed by an outlook on the theoretical enrichment of the concept of walkability.

## 2. Background

During the last decade urban researchers have been interested in how traffic, transportation or air quality are related to physical environmental conditions (and therefore enhance or reduce quality of life). Since the beginning of the millennium, several studies have combined physical environment indicators into different indices (for example, Neighborhood Accessibility Index [8]; built environment index [9]; the 3D’s [10]) and tried to merge them with transportation or traffic data. Mixed land-use, street connectivity and high residential density as urban form indicators seem to be positively related to active transportation [6] and therefore were used to create the so-called Walkability-Index (WAI), which is widely spread in urban planning and active living literature. Since urban research discovered a relationship between urban form and active transportation, public health researchers started to deal with walkability-indices as well. They are mainly interested in enhancing the population’s physical activity volume to reduce non-communicable diseases like obesity, coronary heart diseases and type-2 diabetes. Active living, following Sallis *et al.*, should take place in “four domains of active living”: occupation, household, recreation and transport [11]. The “health enhancing physical activity” (HEPA) recommendation defines at least 150 min of moderate or 75 min of vigorous physical activity during one week in order to reduce health risks. Corresponding to ecological models, researchers in the public-health field focus on different levels to describe and explain person x environment interaction. Bronfenbrenner, one of the originators of socio-ecological or *eco-systematic* approaches, distinguishes between five different environmental subsystems: micro- (the inter-individual interactions), meso- (settings or the sum of inter-individual interaction of a given person), exo- (intraindividual interactions of significant others, e.g., parents, at work site), macro- (norms, traditions, regulations, rules, ideologies) and chrono-systems (normative and non-normative ontogenetic dimensions, e.g. graduation or a severe disease) [12]. Others like Swinburn *et al.* [13] in their work on ecological approaches to analyze obesogenic environments dissect environmental types (physical political and economic) and *environmental sizes*: micro, like settings (e.g., a canteen serving food in schools or at work site) and macro, like sectors (e.g., the transport system to commute to work). Sallis dissects an individual or central level (biology, emotions, self-efficacy, *etc*.), a social or proximal level (friends, family, clubs, *etc*.), an environmental or intermediate level (streets, buildings, accessibility, *etc*.) and a distal level (institutions, policy, culture, *etc*.) [3]. 

Whereas the relationship between walkability and active transportation appears to be quite clear [14,15,16,17], overall physical activity is not always related to the physical environment [17]. So, besides the fact that more research is needed in order to detect associations between the physical environment and physical activity, some scholars argue that future research agendas need to enhance overall assessments with domain-specific physical activity assessments. Environmental and infrastructural influences on physical activity are mainly investigated in the US and in Australia [14,18]. In Europe only a small number of studies have focused on environmental determinants of physical activity, although international findings are certainly also relevant for European settings. In summary, we found large agreement on the following points:

▪ People living in high density areas with a well-connected street network and mixed land use are more likely to walk or cycle to destinations in their neighborhood;
▪ People living near parks or recreational areas are more likely to walk;
▪ People indicating their streets and sidewalks as safe are more likely to walk and to bike.

Overall, we take it from the literature that: (1) environmental influences are significant determinants of physical activity, and (2) walkability seems to be a promising concept to measure the influence of urban areas on health behavior and active lifestyles.

## 3. Methodology

### 3.1. Walkability-Index (WAI)

The WAI we have used in our study was developed by Frank *et al.* [6] and is recommended to calculate walkability by the International Physical Activity and the Environment Network (IPEN). The calculation procedure is implemented in an ArcGIS toolbox available to the general public on the Internet [19]. For reasons of comparability we have adopted the methodological approach as far as possible, using the most detailed datasets available for the Stuttgart region. The resulting index is a combination of multiple criteria that measure aspects of walkability (see [6,18] for more detail):

▪ The *connectivity index* or *intersection density* measures the number of walkable intersections of road per square kilometer. The resulting values show which areas in the city are more interconnected than others, indicating where the layout of roads allows for more pedestrian mobility than elsewhere. The data used here comes from a commercial geodata vendor (*infas Geodaten*) that distributes the *Teleatlas multinet* road network format (*geostreet+*, 2010 version). It contains all road classes, including footpaths.
▪ (*Shannon’s*) *entropy index* is a measure for the quantification of the level of mixed land uses within an area. The assumption here is that the higher the mix of land uses, the more destinations can be reached by foot – thus making the area more walkable. This assumption can only be tested precisely with high-resolution land-use layering. The one used here uses the most detailed digital land-use data available in Germany, which is the cadastral database. The land use classes of the cadaster terminology are mapped to the eight categories required by the software (see Table A1).
▪ The *FAR index* or *floor area ratio* looks at the intensity of shopping opportunities in the city, not only in terms of commercial land use, but also in terms of the retail floor space available. If there are high levels of retail floor space in a commercial land use zone then the shopping opportunities can be expected to be more pedestrian-friendly. A critical aspect here is the land use: the index can be applied to mono-functional commercial areas as well as to mixed-use zones. In both cases higher values indicate a more pedestrian-friendly environment. The data used for this indicator comes once more from the cadastral database, which contains the building blueprints and a function attribute for each building. All buildings with commercial floor space have been included (*“Wirtschaftsgebäude”*, *“Wohn-und Wirtschaftsgebäude”*). Since the actual floor space is not known, the blueprint size of the buildings is being used as the nearest approximation available (this is a generalization that has also been accepted by [18] in their model implementation of WAI).
▪ The *household density index* is probably the easiest one to implement: it simply divides the number of households by the land use category “living”. Higher values are assumed to be more pedestrian friendly than lower density values. The number of households is derived from a geomarketing dataset distributed by infas Geodaten (*“Wohnquartiere”*) for 2010. The spatial units for this dataset have also been chosen as urban area sub-districts. They contain approximately 500 households each and can be labeled as “neighborhoods” since they have similar land use characteristics.

**Figure 1:**
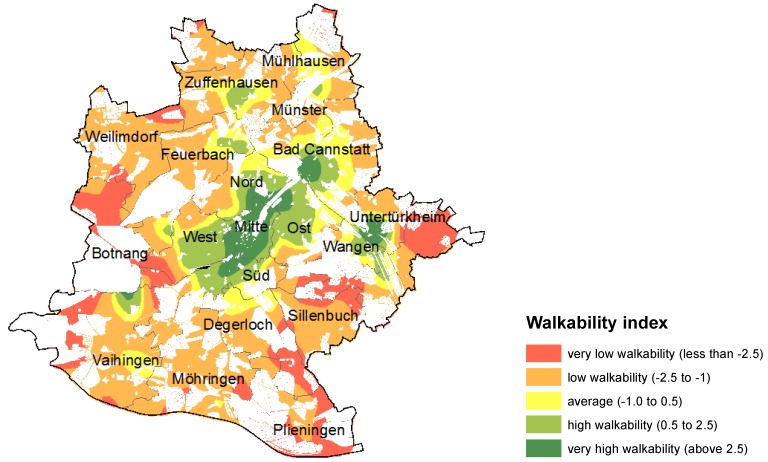
Walkability index (WAI) for the City of Stuttgart.

Notes: Data sources: geostreet+ 2010; infas Wohnquartiere 2010; Automatisiertes Liegenschaftskataster (ALK 2008); Land use: Amtliches-Topographisch-Kartographisches Informationssystem (DLM25 2011). Cartography: Heat map using the ArcGIS 10 Geostatistical Analyst method “diffusion kernel” (500 m bandwidth), using uninhabited blocks as barriers and the number of households as weights.

The final score of WAI is a simple aggregation of the standardized indicators listed above, with a double weighting for the connectivity index. The implementation in Figure 1 shows a generalized heat map for WAI in Stuttgart, based on the spatial level of neighborhoods. This generalization is necessary since the neighborhood level is too heterogeneous at the city level and the heat mapping approach helps to identify the underlying patterns. Not surprisingly, “very high to high” walkability can be found in the city center and in main urban sub-centers (Bad Cannstatt, Untertuerkheim/Wangen, Zuffenhausen, the university campus north of Vaihingen). The map also highlights the locations of “low to very low” walkability, which are mainly at the outskirts of the suburban areas, with a slight concentration of very low walkability in the South.

Overall, WAI produces plausible results in terms of the variation of high and low walkability levels throughout the city. We will later test this interpretation using several questionnaire items from a household survey (see Section 3.3). We conclude that the WAI successfully captures the variations of urban form that seem to be relevant for walkability. However, the methodological approach has weaknesses in its generalization of land use classes for the land use mix measured by the Shannon’s entropy index, particularly since the inclusion of industrial land into the land use mix actually improves the measured walkability. In our view, land use mix cannot be a proxy for actual walkability since it does not give any information about actual destinations people walk to. This issue has been picked up by the concept of the Walk Score described in the next section.

### 3.2. Walk Score

The Walk Score approach to measure walkability has been developed by a commercial company of the same name in Seattle, United States of America. The objective is to measure the friendliness towards walking of a specific address based on the proximity of important amenities for everyday life like grocery shops and supermarkets, but also for cultural and entertainment activities like cinemas, restaurants, etc. It currently works as an Internet platform [20] for addresses in the Unites States, Canada, Australia and New Zealand. For other parts of the world it works technically, but the databases behind it are often insufficient to produce valid and reliable empirical results. 

**Figure 2:**
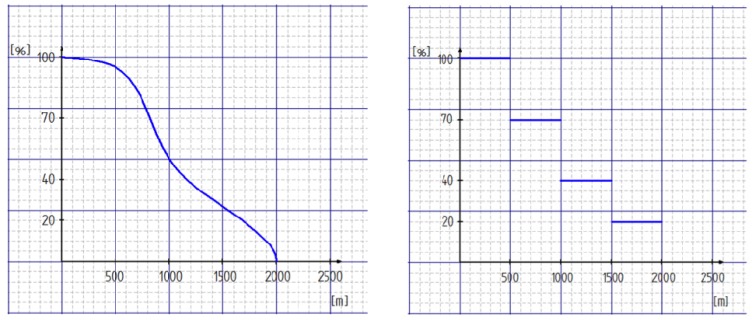
Distance decay function for the calculation of the Walk Score (**left**) and the simplified adaptation in terms of distance bands (**right**).

The original Walk Score implementation uses the facilities and weightings in the left column of Table 1 (in brackets is the number of facilities that are used for an average distance calculation; if there are additional brackets, then a weighting is used.). The weighting reflects the importance of a certain facility for everyday life. For example, grocery shopping (3 points) in the vicinity of residence is more important than entertainment (1 point). For the categories restaurants/bars, shopping, and cafés it is acknowledged that the variety and choice of options plays an important role. For this reason, the total number of points is divided amongst the different options. The company “Walk Score” calculates the values based on data from Google, Education.com, OpenStreetMap and Localize, using the distance between an address entered to the addresses of facilities of each category. The calculation of points uses a distance decay function (see Figure 2), the assessment then transforms values to a scale of “0” to “100”, where “100” is the best result and represents a point score of “15”.

In this context it should be mentioned that the Walk Score approach has been frequently criticized for its applicability when data sources are sparse and highly generalized. For example, the original Walk Score does not differentiate between small corner shops that sell groceries and a fully equipped supermarket. Our implementation for Stuttgart overcomes this problem since it works with key service amenities (called “errands” in the Walk Score literature) extracted from public German business directories. Other than that we follow the original Walk Score approach as closely as possible. The datasets were chosen for their relevance in everyday life for a broad range of activities, including supermarkets, restaurants/bars, banks, social institutions like schools, and recreational facilities like public parks and entertainment facilities. The Walk Score calculated for Stuttgart uses ten different categories, which were adopted from the US implementation. The categories and weightings are shown in Table 1.

The distance to the selected facilities serves as the basis for Walk Score calculations. The maximum search radius is two kilometers network distance along a TomTom (formerly Teleatlas) street network. Figure 2 shows the adaptation of the Walk Score distance decay function that has been simplified to distance bands in this implementation. The reason for this methodological modification was the software limitations in GIS that did not allow the use of functions in a straightforward manner.

**Table 1:** Categories of errands used for Walk Score calculation in this study (adopted from [20]).

Point system for the Walk Score calculation ([number of points], *n*{weightings})	
grocery stores including supermarkets [3] restaurants/bars [3] = {0.75, 0.45, 0.25, 0.25, 0.225, 0.225, 0.225, 0.225, 0.2, 0.2} shopping [2] = {0.5, 0.45, 0.4, 0.35, 0.3} schools [1]	bakery/cafés [2] = {1.25, 0.75} entertainment [1] banks [1] recreation/parks [1] books [1]

**Figure 3:**
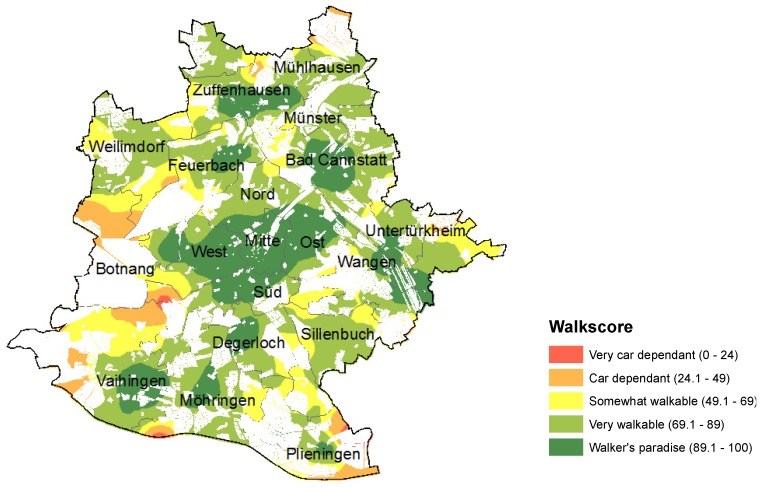
Walk Score for Stuttgart.

*Notes*: Data sources: geostreet+ 2010; infas Wohnquartiere 2010; Automatisiertes Liegenschaftskataster (ALK 2008); Parks: Amtliches-Topographisch-Kartographisches Informationssystem (DLM25 2011); Yellow Pages (other facilities). Cartography: Heat map using the ArcGIS 10 GeostatisticalAnalyst method, diffusion kernel (500 m bandwidth), using uninhabited blocks as barriers and the number of households as weights.

We computed the Walk Score for 2,259 household addresses in the city, representing 656 inhabited neighborhood blocks. Figure 3 shows the result for the city of Stuttgart, cartographically generalized with heat map functions. For each point location the Walk Score for the 500 meter area around it is computed from all Walk Score values within it, using a diffusion kernel function and excluding uninhabited blocks. The legend and colors clearly indicate that the city of Stuttgart is by and large very walkable and that there are few areas in the periphery (mainly in the east, but also in a second ring of suburban settings around the center) that can be seen as rather car dependent (red and yellow colors). 

In contrast to WAI, this measure has the advantage that it can be compared to other regions easily, provided that similar data is being used. It is not a relative assessment like WAI and has therefore the potential to inform about walkability in a homogeneous and uniform way. 

### 3.3. Statistical Analyses

Geo-referenced household survey data collected by the Regional Authority (Verband Region Stuttgart [21]) from 2009 to 2010 was used to assess the effects of WAI and Walk Score on active transportation. The survey design was based on the standards of a weekly household survey according to the survey design developed by the Germany Mobility Panel (*Deutsches Mobilitätspanel MOP*) with an aim to cover 4,000 households in two survey periods in spring and fall. Participants were selected with a probability proportional to size-method, *i.e.*, a register-based selection method with uneven selection probabilities. The Walk Score was recalculated for each of these households with its exact geographic coordinates, yielding a maximum score of 13 accessibility points (out of 15, see Table 1). Apart from trip characteristics during one week (length, origin and destination, trip purpose, *etc.*), the database also provides the link to socio-demographic information (age, sex, income, *etc.*) and other mobility-related information. In our analyses we included all trips of respondents within a 1.6 km (1.5 miles, according to [8]) network buffer around their home that started and/or ended at their home. Further we included only trips for transportation purposes done by people older than 18 years as well as people with no mobility constraints (e.g., visual impairments).

Based on the data we calculated three variables, which, taken from the literature, depend on the WAI with respect to the Walk Score: (1) “Number of walking trips for transport” is the number of walking trips for transportation purposes in the neighborhood (within a 1.6 km network buffer around the subjects’ homes) within one week. (2) “Walked distance for transport” is the walked distance for transportation purposes within one week given in km, and (3) “Minutes of walking for transport” is the duration of walking trips for transportation purposes within one week.

The final sample consisted of 1,871 residents living in 491 neighborhoods (block groups). Demographic variables for the sample are shown in Table 2. The average walked distance for transportation purposes in the neighborhood in one week was 3.38 km and the average minutes of walking for transportation per week was 64. The average number of walking trips for transportation in the neighborhood was 5.7.

We used linear regression to predict the dependent (criterion) variables “minutes of walking” and “walked distance” using the WAI as well as the Walk Score as predictors. For “number of walking trips for transport” we used the generalized linear model because of its poisson-distribution as a count variable. 

We conducted the linear regression in two steps: in the first step we entered three socio-demographic variables (model without WAI and Walk Score) and in the second step we entered the WAI respectively the Walk Score (full model) to assess their contribution to the explained variance in the criteria (for a similar procedure see [22]). 

**Table 2:** Model variables (*n* = 1,871) and sample characteristics.

Dependent variables	%	Median	Mean	SD	Range
Number of walking trips for transport per week		4.0	5.7	5.1	1–37
Minutes of walking for transport per week		42	64	67	2–658
Walked distance for transport (in km) per week		2.28	3.38	3.33	0.01–23.8
Independent variables					
Walkability-Index (WAI)		−0.81	−0.16	3.15	−4.7–21.3
Walk Score		81.0	79.2	16.1	16.7–100
*Demographic and socioeconomic covariates*					
Sex (male)	42.8				
Age (in years)		55.0	54.1		18–92
Monthly household income		2 ^a^			

^a^ this corresponds to 1.500 to 2.999 € per month.

Table 3 and Table 4 summarize our results: the associations between the WAI respectively the Walk Score and the criterion variables “walked distance for transport” and “walked minutes for transport” are significant, but the adjusted *R*^2^ are very small. Based on the adjusted *R*^2^ the WAI explains an additional variance of 0.6% in walked distance for transport and the Walk Score 1.9% (see Table 3). For minutes of walking for transport the WAI explains an additional variance of 0.7% and the Walk Score 2.3% (see Table 4). As expected, WAI and Walk Score were positively related to walking for transport. But the common shared variances of the predictors and the criterions were lower than in comparable studies [22]. 

A one unit change in the WAI would therefore increase walked distance for transport by 0.091 km and a one unit change in the Walk Score would increase walked distance for transport by 0.03 km. Minutes of walking for transport is increased by 0.649 min by Walk Score and by 1.841 min by WAI.

Among the two criterion variables WAI and Walk Score predicted the “minutes of walking for transport” best whereas the Walk Score could add more explanation of variance than the WAI.

The results of the poisson regression analysis point into the same direction. We found significant associations between WAI respectively Walk Score and walking trips per week but regression coefficients are rather small. A one unit change in WAI would increase the number of walking trips by factor 1.04 and a one unit change in the Walk Score would increase the number of walking trips by factor 1.01. Table 5 gives an overview of the result of the poisson-regression models. 

**Table 3:** Regression models for walked distance for transport per week with and without WAI and Walk Score.

Independent Variables	Unstandardized coefficients		Standardized coefficients	*t*	Sig.	Partial Correlation
	B	SE	Beta			
Constant	4.074	0.396	--	10.292	0.001	--
Income	−0.166	0.115	−0.034	−1.453	0.146	−0.034
Sex	−0.621	0.155	−0.092	−4.007	0.001	−0.092
Age	−0.001	0.005	−0.005	−.197	0.844	−0.005
WAI	0.091	0.024	0.086	3.700	0.001	0.085
Constant	1.446	0.595	--	2.428	0.015	--
Income	−0.119	0.114	−0.024	−1.038	0.299	−0.024
Sex	−0.614	0.154	−0.091	−3.991	0.001	−0.092
Age	0.002	0.005	0.009	0.364	0.716	0.008
Walk Score	0.030	0.005	0.144	6.220	0.001	0.143
**Walked distance for transport**		**Model without WAI**	**Full model**	**Model without Walk Score**		**Full model**
*R*^2^		0.010	0.018	0.010		0.030
Adjusted *R*^2^		0.009	0.015	0.009		0.028
*R*^2^ change		0.010	0.007	0.010		0.020
*F* change		6.479	13.693	6.479		38.688
Sig. of *F* change		0.001	0.001	0.001		0.001
d*f*		3, 1,867	1, 1,866	3, 1,867		1, 1,866

**Table 4:** Regression models for minutes of walking for transport per week with and without WAI and Walk Score.

Independent Variables	Unstandardized coefficients	Standardized coefficients	*t*	Sig.		Partial Correlation
	B	SE	Beta			
Constant	67.190	7.929	--	8.473	0.001	--
Income	−5.958	2.294	−0.060	−2.597	0.009	−0.060
Sex	−15.269	3.103	−0.112	−4.920	0.001	−0.113
Age	0.316	0.093	0.079	3.406	0.001	0.079
WAI	1.841	0.490	0.086	3.756	0.001	0.087
Constant	9.844	11.906	--	0.827	0.408	--
Income	−4.866	2.283	−0.049	−2.131	0.033	−0.049
Sex	−15.142	3.077	−0.111	−4.922	0.001	−0.113
Age	0.375	0.092	0.094	4.053	0.001	0.093
Walk Score	0.649	0.096	0.155	6.759	0.001	0.155
**Minutes of walking for transport**		**Model without WAI**	**Full model**	**Model without Walk Score**		**Full model**
*R*^2^		0.024	0.031	0.024		0.047
Adjusted *R*^2^		0.022	0.029	0.022		0.045
*R*^2^ change		0.024	0.007	0.024		0.001
*F* change		15.118	14.108	15.118		45.685
Sig. of *F* change		0.001	0.001	0.001		0.001
d*f*		3, 1,867	1, 1,866	3, 1,867		1, 1,866

**Table 5:** Poisson regression models for predictors of number of walking trips per week

Explanatory variables	WAI				Walk Score			
	B (SE)	Exp(B)	Wald test (d*f*)	*p*	B (SE)	Exp(B)	Wald test (d*f*)	*p*
**Constant**	1.51 (0.04)	4.54	1,735.51	0.001	0.59 (0.07)	1.80	73.78	0.001
**Income**								
1–1,499€	0.07 (0.03)	1.07	5.13	0.023	0.03 (0.03)	1.03	1.03	0.310
1,500–2,999€	0.10 (0.02)	1.10	18.82	0.001	0.10 (0.02)	1.10	19.58	0.001
over 3,000 (ref.)								
**Sex**								
female	0.21 (0.02)	1.24	113.75	0.001	0.21 (0.02)	1.24	111.21	0.001
male (ref.)								
**Age**	0.001 (0.00)	1.00	1.65	0.199	0.002 (0.00)	1.00	6.72	0.010
**WAI/Walk Score**	0.04 (0.00)	1.04	178.81	0.001	0.01 (0.00)	1.01	281.03	0.001

## 4. Discussion and Conclusion

Our findings are consistent with previous research on walkability and active transportation [notably in Belgium and Sweden, see for example [17,22,23,24,25,26] and point into the expected direction. Van Dyck *et al.* [24] report that living in a high-walkable neighborhood was associated with the weekly minutes of walking for transportation as well as the accelerometer-based moderate to vigorous physical activity (MVPA) in a sample of 1,166 Belgian adults. Sundquist *et al.* [25] also reported that people living in highly walkable areas showed more minutes of MVPA and walked more often for transportation purposes. Two reviews from 2012 point into the same direction: Van Holle *et al.* [17] as well as Grasser *et al.* [15] illustrated the positive associations between the physical environment and walking for transportation. Although different predictor and criterion variables are being used, the tendency towards more active transportation in more walkable neighborhoods exists. However, the effect size is small. Nevertheless the public health impact is worth mentioning. As Lee *et al.* [27] pointed out recently, even small increases in physical activity can have great public health impact. Thus the estimated gain in life expectancy if physical inactivity was eliminated is expected to be 0.47 years. If it had not been calculated on the whole population base, but on the number of inactive people, life expectancy would increase even more. The likely reason for the small statistical effect size is that walking is a complex individual behavior, not solely determined by the built environment.

Although the results of the regression analyses showed into the expected direction they were somehow unexpected as well. Several studies use distance or duration measures (minutes per week or kilometers per day) to analyze associations between environmental variables and walking behavior. So did we although we think that other dependent variables must be taken into account, as living in high walkable areas should lead to shorter walking distances and shorter travel times. The question remains which dependent variable is best suited for walkability analyses. In further studies, we suggest analyzing physical activity in different domains (e.g., transport or recreation) by data collection methods using accelerometry combined with walking diaries and GPS.

Our results can also be interpreted in another way. The relatively weak statistical associations between urban form characteristics (walkability here) and walking behavior that we found for the city of Stuttgart might be explained by a much lesser “walking suppressive” urban infrastructure than in North America or Australia. Unlike in the USA, German suburbs with very low densities residential streets typically have sidewalks and people can walk safely. Taking the fundamental differences of urban form and transportation infrastructure in Europe and North America into account, walkability might not be a less powerful influencing factor of personal mobility in the European context but more difficult to detect. 

Nevertheless, considering the available methods and their adaptation to a typical German city, there remains a lot of empirical and theoretical work to do. We are convinced that walkability as proposed by the IPEN-Network [28] is a first step towards describing the influences of the built environment on physical activity in a systematic manner. In this context, the Walk Score is a promising new concept to the WAI preferred so far in walkability assessments. 

In addition, we believe that walkability could gain more explanatory impact by adding a more robust theoretical grounding. One of several possible affiliations could be Barker’s concept of behavior setting [29], or more recent theoretical frameworks in environmental gerontology [30]. These frameworks point to the fact that specific environments force necessary and proper behaviors. People feel compelled to act as their built and social environment prompts them to do. Neighborhoods also entail a lot of emergent norms. Belonging to the neighborhood most often means adjusting behavior to these norms. Smedley and Syme [31] have pointed out in the *American Journal of Health Promotion*, that interventions to promote health should always address the people’s environment. This would support to motivate people to be more active and to stick to this healthy behavior. In this context, environment is both the social and the built environment.

For the purpose of more efficient interdisciplinary research, it seems worthwhile to integrate descriptions of the environment with theoretical frameworks of walkability. This will avoid crude “empiristic” approaches, which only deliver data but no content. Social-ecological frameworks work best here. Apart from special aspects, they have the following common paradigms:

▪ Person and environment are mutually connected. People do not only react to their environment, but they act as agents of their own needs and personal strivings. They often use the environment to fulfill their needs or to train their skills and abilities;
▪ Environment is more than physical, it is also subjective and most often a socially shared environment.

Following these two paradigms, it is necessary to start studies in the natural setting that follow an ecological approach when collecting data and studying people’s behavior outside a lab. Looking into the literature and the traditions in public health and health sciences, socio-ecological approaches are well established (e.g., [32]). For example, Wahl, Iwarsson und Oswald [30] deal with the influences of built and social environment on successful aging. In old age, more than in younger life, built environment is critical for active living. It can be a barrier or it can enable living a self-determined and autonomous life. It can also help to satisfy subjective needs like social affiliation and add to quality of life. In a descriptive model proposed by Wahl *et al.* [30], two core processes determine older peoples’ behavior. One process is identity, described as belonging to a social group and to a specific environment. Belonging, a feeling, is a cornerstone of the social-identity or the social-self people have. In social psychology, Neisser [33] points to the fact that spaces we behave in deliver information about who we are. The concept of walkability describes spaces, but does not deliver identity-relevant information. The second process in the model of Wahl and colleagues is agency, which is a conscious and targeted act to form the environment to satisfy own needs and reach personal goals. There are some results in gerontology showing that this process loses its importance when people grow older. 

In socio-ecological approaches, the interface between person and environment is most often an emergent person-by-environment (P-E fit) agent. This is because skills and abilities are only significant in corresponding environments. Vice versa, environmental features are only useful in conjunction with a person’s ability to use them. Walkability as an example is only useful in association with the person’s ability and motivation to walk.

We took environmental gerontology as an example here. Looking to other disciplines like economy or philosophy there are comparable approaches focusing the P × E interaction. Another example is the minded capability approach by Nussbaum and Sen [34], which looks at people’s capability of realizing their subjective valued goals. People not only need means and resources but also conversion factors (personal, social and environmental) to maximize their options and freedom of action. There are inequalities in opportunity across nations, social groups and individuals. Sen and followers ask what the opportunities and barriers are that allow people to live self-determined and autonomous lives. Related to our topic here, the capability approach could enrich the usefulness of the walkability concept. A high walkability enhances older people’s opportunities to be mobile. While going on errands they can invest in social contacts. This is a significant opportunity to live a self-determined life. In contrast, low walkability restrains and personal striving is hindered.

## Appendix

**Table A1:** Land use data mappings.

Land use (German cadastral terminology)		Land use categories (and abbreviations) required by the walkability toolbox	
German	English		
Ackerland	Farmland	Other	O
Bach	Stream	Water	W
Bahngelände	Railway property	Other	O
Bauplatz	Building lot	Living	L
Betriebsfläche Abbauland	Asset area for mining and extraction	Industrial	I
Betriebsfläche Entsorgungsanlage	Asset area for waste disposal	Industrial	I
Betriebsfläche Halde	Asset area for mining waste	Industrial	I
Betriebsfläche Lagerplatz	Asset area for storage	Industrial	I
Brachland	Fallow	Other	O
Campingplatz	Campground	Recreational	R
Flugplatz	Airport	Other	O
Fluß	River	Water	W
Friedhof	Cemetery	Recreational	R
Gartenland	Garden land	Other	O
Gebäude- und Freifläche Erholung	Built-up area for recreation	Recreational	R
Gebäude- und Freifläche Gewerbe und Industrie	Built-up area for business and industry	Commercial	C
Gebäude- und Freifläche Handel und Wirtschaft	Built-up area for retail and commerce	Services	S
Gebäude- und Freifläche Land- und Forstwirtschaft	Built-up area for agriculture and forestry	Other	O
Gebäude- und Freifläche Öffentliche Zwecke	Built-up area for public use	Institutional	T
Gebäude- und Freifläche Wohnen	Built-up area residential	Living	L
Gebäude- und Freifläche zu Entsorgungsanlagen	Built-up area for waste disposal	Industrial	I
Gebäude- und Freifläche zu Versorgungsanlagen	Built-up area for public services	Industrial	I
Gehölz	Grove	Other	O
Graben	Ditch	Other	O
Grünanlage	Park	Recreational	R
Grünland	Grassland	Other	O
Hafen	Port	Water	W
Historische Anlage	Monument	Other	O
Kanal	Channel	Water	W
Laubwald	Deciduous forest	Other	O
Mischwald	Mixed forest	Other	O
Parkplatz	Parking lot	Other	O
Platz	Plaza	Other	O
Schiffsverkehr	Shipping traffic	Water	W
See	Lake	Water	W
Sportfläche	Sports area	Recreational	R
Straße	Street	Other	O
Teich	Pond	Water	W
Übungsgelände	Exercise area	Other	O
Unland	Wasteland	Other	O
Weg	Lane	Other	O
Weingarten	Vineyard	Other	O
